# Liquid biopsy using plasma proteomics in predicting efficacy and tolerance of PD-1/PD-L1 blockades in NSCLC: a prospective exploratory study

**DOI:** 10.1186/s43556-025-00291-6

**Published:** 2025-07-15

**Authors:** Yuan Gao, Fei Qi, Wenhao Zhou, Peng Jiang, Mingming Hu, Ying Wang, Congcong Song, Yi Han, Dongdong Li, Na Qin, Hongmei Zhang, Haitao Luo, Tongmei Zhang, Hongxia Li

**Affiliations:** 1 General Department, Beijing Chest Hospital, Capital Medical University, Beijing Tuberculosis and Thoracic Tumor Research Institute, Beijing, China; 2 Laboratory for Clinical Medicine, Capital Medical University, Beijing, China; 3 Shenzhen Engineering Center for Translational Medicine of Precision Cancer Immunodiagnosis and Therapy, YuceBio Technology Co, Ltd, Shenzhen, China; 4 Cardiothoracic Surgery Department, Affiliated Hospital of Shaanxi University of Chinese Medicine, Shaanxi University of Chinese Medicine, Shaanxi, China; 5 School of Life Sciences, Anhui Medical University, Anhui, China; 6 Outpatient Department, Beijing Chest Hospital, Capital Medical University, Beijing Tuberculosis and Thoracic Tumor Research Institute, Beijing, China

**Keywords:** Non-small cell lung cancer, Immune checkpoint inhibitor, Plasma proteomics, Efficacy, Risk score

## Abstract

Immune checkpoint inhibitors (ICIs) show limited efficacy in non-small cell lung cancer (NSCLC), highlighting the need for predictive biomarkers. Here we prospectively analysed serial plasma samples from 34 ICI-treated advanced NSCLC patients (plus 30 validation samples) using the Olink Immuno-Oncology panel. We assessed dynamic proteomic changes associated with ICI efficacy and immune-related adverse events (irAEs), and developed a prognostic model. Following ICIs, 42/92 proteins significantly elevated upon ICI treatment (*p* < 0.05). Baseline levels of CD28, CXCL10, and TNFSF14, and increased CD40L post-treatment, correlated with inferior response. Baseline IL-4, IL-13 and increased GZMA post-treatment were associated with irAE occurrence. Using LASSO-Cox regression, we established an Immunosuppressive Signature of Combined Resistance Elements (I-SCORE) model based on eight plasma proteins (CCL23, ARG1, CD83, ADA, CXCL10, TNFSF14, CD28, GZMA). I-SCORE demonstrated strong predictive power for overall survival (12-month AUC = 0.94), progression-free survival (12-month AUC = 0.75), and treatment response (AUC = 0.62). Furthermore, a high I-SCORE was demonstrated to reflect an inflammatory and immunosuppressive phenotype, showing positive linear relationships with plasma IL-6, IL-8, IL-10, and monocyte count, and negative relationships with IL-33 and active T-cell proportion. Our study identifies I-SCORE, derived from plasma proteomics, as a promising integrated biomarker for predicting ICI outcomes in NSCLC. It suggests targeting specific proteins or the associated immunosuppressive microenvironment might enhance immunotherapy efficacy.

**Supplementary Information:**

The online version contains supplementary material available at 10.1186/s43556-025-00291-6.

## Introduction

Immune checkpoint inhibitor (ICI) targeting PD-1 or PD-L1 have achieved huge clinical success in treating advanced non-small cell lung cancer (NSCLC) [1–6]. However, the effiacay of ICI therapy is limited and only a small subset of patients benefits from it. Some do not respond to therapy (primary resistance/refractory) and some responders relapse after initial response (acquired resistance) [7]. The mechanisms underlying primary and acquired resistance to ICI are not fully understood. At present, PD-L1 expression, tumor mutation burden (TMB), and tumor infiltrating lymphocytes (TILs) were reported as possible biomarkers for distinguishing responders and non-responders in NSCLC and other solid tumors [8–10]. However, existing biomarkers remain largely restricted to either tumor-intrinsic or extrinsic factors with suboptimal predictive power. Moreover, the limited accessibility and invasive nature of tumor biopsies further constrain their clinical utility. Thus, identifying novel predictive biomarkers represents an unmet clinical need.

Given the dynamic interaction between tumor and microenvironment, multidimensional biomarker panels incorporating both tumor- and host-derived parameters are expected to demonstrate superior predictive accuracy versus single factor isolated from either tumor-intrinsic or extrinsic sources. Plasma, which contains cytokines and resultants released from tumor, immune cells and their interaction, may provide comprehensive valuable information on ICI treatment [11]. Moreover, as a non-invasive liquid biopsy, plasma detections allow dynamic monitoring during the treatment course overcoming tumor spatial and temporal heterogeneity. At present, plasma proteomics has been increasing used in biomedical research providing complementary information to genomics and transcriptomics [12]. Based on enzyme-linked immunosorbent assays (ELISA), a recent study profiled the plasma proteomics of NSCLC patients and identified CXCL8 and CXCL10 as biomarker for inferior response to ICI [13].

Olink biosecence, one advanced technology for proteomics, has the advantage of simultaneous quantification of a large number of proteins with only small volumes of sample [14–17]. Plasma Olink proteome sequencing provide comprehensive information on tumor intrinsic feature, immune status and their mutual interactions, and therefore may be a reliable liquid biopsy in biomarker exploration [13, 18–20]. A large-scale plasma proteome analysis screened leukemia inhibitory factor (LIF) as a new peripheral blood protein marker for predicting ICI efficacy in multiple cancers.[20] Using the Olink Immuno-Oncology panel (92 proteins) with proximity extension assays (PEA), Gao demonstrated the associations between IL-8, TIE2 and HGF and prognosis among patients with esophageal cancer [19]. In another study of 18 patients with stage III NSCLC receiving concurrent chemoradiotherapy, CR2 and IFNGR2 were identified as positive prognostic biomarkers and treatment-induced changes of CXCL10 and IL-10 as negative predictive biomarkers [21].

In this study, we used Olink Immuno-Oncology panel with PEA to explore the dynamic changes in plasma protein upon ICI treatment and surrogate predictive biomarkers for NSCLC. We found that proteins involving in cytokine-cytokine receptor interaction, chemokine signaling pathway and immune response were significantly up-regulated after ICI therapy. Moreover, several proteins were identified to be associated with remission, toxicity and survival outcome of ICI therapy. Furthermore, using Least Absolute Shrinkage and Selection Operator (LASSO) + Cox regression, we established an immunosuppressive signature of combined resistance elements (I-SCORE) model based on eight predictive proteins, which was demonstrated to have quite good predictive values on both response and survivals for ICI treatment. High I-SCORE implied an inflammatory and suppressive status of patients with inferior prognosis.

## Result

### Patient cohort and plasma samples

**Fig. 1:**
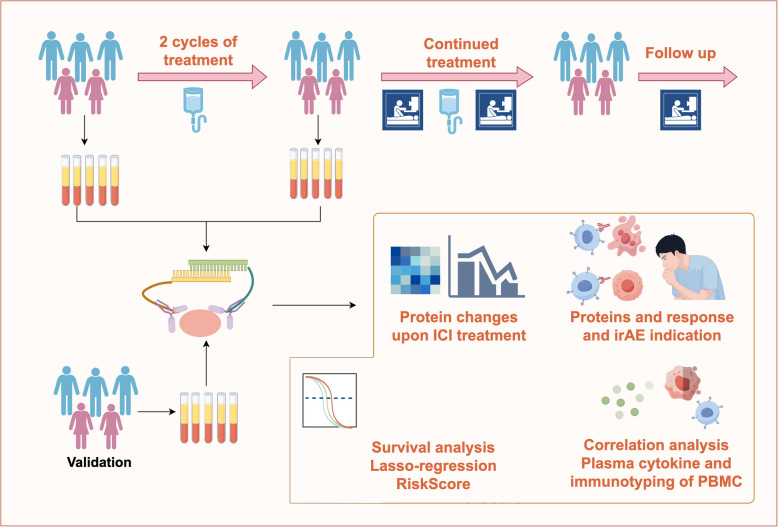
Study schema of this study

This study consisted two patient cohorts including the study cohort (Cohort 1) and the validation cohort (Cohort 2). Study schema was presented in Fig. 1. A total of 34 eligible patients with stage IIIB-IV NSCLC who received ICIs between August 2020 and June 2023 at Beijing Chest Hospital were enrolled in Cohort 1. Patient characteristics were summarized in Table 1. The median age was 62.6 (range, 33–79) years and the male to female ratio was 4.67:1. Overall, 29.4% of patients were squamous carcinoma and 70.6% were non-squamous NSCLC. Majority of patients had no prior systemic treatment (29/34, 85.3%) before enrollment and 5 patients harboring EGFR mutation who failed EGFR-TKI were also enrolled. All enrolled patients received PD-1 (n = 30, 88.2%) or PD-L1 (n = 4, 11.8%) blockades among which 28 cases (82.3%) had additional chemotherapy. An additional 30 cases receiving ICI treatment from 2023 to 2024 were reviewed as the validation cohort (Cohort 2). The baseline characteristics were well balanced between the two cohorts of patients (Table 1).

**Table 1:** Patient baseline characteristics

Characteristics	Cohort 1				Cohort 2				*p*
	**Total**	**Response**			**Total**	**Response**			
		**PR**	**SD + PD**	***p***		**PR**	**SD + PD**	***p***	
**Age**				1.000				0.694	0.102
** < 60 years**	13 (38.2)	5 (38.5)	8 (38.1)		9 (30.0)	5 (35.7)	4 (25.0)		
** ≥ 60 years**	21 (61.7)	8 (61.5)	13 (61.9)		21(70.0)	9 (64.3)	12 (75.0)		
**Gender**				0.021					0.634
**Male**	28 (82.3)	8 (61.5)	20 (95.2)		26 (86.7)	13 (92.9)	13 (81.3)		
**Female**	6 (17.7)	5 (38.5)	1 (4.8)		4 (13.3)	1 (7.1)	3 (8.7)		
**Pathology**				0.815				0.464	0.147
**ADC**	21 (61.8)	9 (69.2)	12 (57.1)		15 (50.0)	6 (42.9)	9 (56.3)		
**SCC**	10 (29.4)	3 (23.1)	7 (33.3)		15 (50.0)	8 (57.1)	7 (43.7)		
**NSCLC-un**	1 (2.9)	1 (7.7)	1 (4.8)		0	0	0		
**LCNEC**	1 (2.9)	0	1 (4.8)		0	0	0		
**Smoking**				1.000				1.000	0.733
**No**	26 (76.4)	10 (76.9)	16 (76.2)		24 (80.0)	11 (78.6)	13 (81.3)		
**Yes**	8 (23.6)	3 (23.1)	5 (23.8)		6 (20.0)	3 (21.4)	3 (8.7)		
**T-stage**				0.487				0.789	0.662
**1–2**	12 (35.3)	4 (30.8)	8 (38.1)		9 (30.0)	5 (35.7)	4 (25.0)		
**3–4**	22 (64.7)	9 (69.2)	13 (61.9)		21 (70.0)	9 (64.3)	12 (75.0)		
**N stage**				0.345				0.950	0.672
**0**	4 (11.8)	2 (15.4)	2 (9.5)		5 (16.7)	2 (14.3)	3 (18.8)		
**1**	3 (8.8)	2 (15.4)	1 (4.8)		3 (10.0)	2 (14.3)	1 (6.2)		
**2**	13 (38.2)	6 (46.2)	7 (33.3)		14 (46.7)	6 (42.9)	8 (50.0)		
**3**	14 (41.2)	3 (23.1)	11 (52.4)		8 (26.6)	4 (28.5)	4 (25.0)		
**M stage**				0.780				0.396	0.355
**0**	5 (14.7)	2 (15.4)	3 (14.3)		5 (16.7)	2 (14.3)	3 (18.8)		
**1a**	7 (20.6)	2 (15.4)	5 (23.8)		12 (40.0)	4 (28.6)	8 (50.0)		
**1b**	5 (14.7)	1 (7.7)	4 (19.0)		4 (13.3)	2 (14.3)	2 (12.5)		
**1c**	17 (50.0)	8 (61.5)	9 (42.9)		9 (30.0)	6 (42.8)	3 (18.7)		
**TNM stage**				1.000				1.000	0.829
**IIIB**	5 (14.7)	2 (15.4)	3 (14.3)		5 (16.7)	2 (14.3)	3 (18.8)		
**IV**	29 (85.3)	11 (84.6)	18 (85.7)		25 (83.3)	12 (85.7)	13 (81.2)		
**EGFR status**				0.056				0.087	0.570
**Mutation**	5 (14.7)	0	5 (23.8)		3 (10.0)	0	3 (18.8)		
**Wild type**	29 (85.3)	13 (100)	16 (76.2)		27 (90.0)	14 (100)	13 (81.2)		
**PD-L1 TPS**				0.248				0.102	0.688
** < 1%**	7 (20.6)	4 (30.8)	3 (14.3)		5 (20.0)	4 (28.6)	1 (6.2)		
** ≥ 1%**	27 (79.4)	9 (69.2)	18 (85.7)		25 (80.0)	10 (71.4)	15 (93.8)		
**ICI line**				0.370				1.000	0.101
**1st line**	28 (82.3)	12 (92.3)	16 (76.2)		28 (93.3)	13 (92.9)	15 (93.7)		
** ≥ 2nd line**	6 (17.7)	1 (7.7)	5 (23.8)		2 (6.7)	1 (7.1)	1 (6.3)		
**ICI**				0.274				1.000	0.810
**Anti-PD-1**	30 (88.2)	10 (76.9)	20 (95.2)		23 (76.7)	11 (78.6)	12 (75.0)		
**Anti-PD-L1**	4 (11.8)	3 (23.1)	1 (4.8)		7 (23.3)	3 (21.4)	4 (25.0)		
**Therapy**				0.653				0.467	0.109
**ICI**	6 (17.7)	3 (23.1)	3 (14.7)		1 (3.3)	1 (7.1)	0		
**ICI + CT**	28 (82.3)	10 (76.9)	18 (85.7)		29 (96.7)	13 (92.9)	16 (100)		

Abbreviations: *ADC* Adenocarcinoma, *CT* Chemotherapy, *PR* Partial response, *SD* Stable diseases, *PD* Progression of disease, *NSCLC* Non-small cell lung cancer, *NSCLC-un* NSCLC-unclassified, *LCNEC* Large cell neuroendocrine carcinoma, *SCC* Squamous carcinoma, *ICI* Immune checkpoint inhibitor, *PD-1* Programmed cell death protein 1, *PD-L1* Programmed cell death-Ligand 1, *WT* Wild type, *MT* Mutation.

A total of 58 pre-treatment and post-treatment plasma samples were collected from patients in Cohort 1 and 30 pre-treatment plasmas were extracted from patients in Cohort 2. Olink proteomic assays were performed using a 92-Immuno-Oncology panel (Table S1) to assess dynamic change of plasma proteins and predictive biomarkers for ICI therapy.

### Treatment response, survival and immune related adverse event (irAE) of ICI treatment

**Fig. 2:**
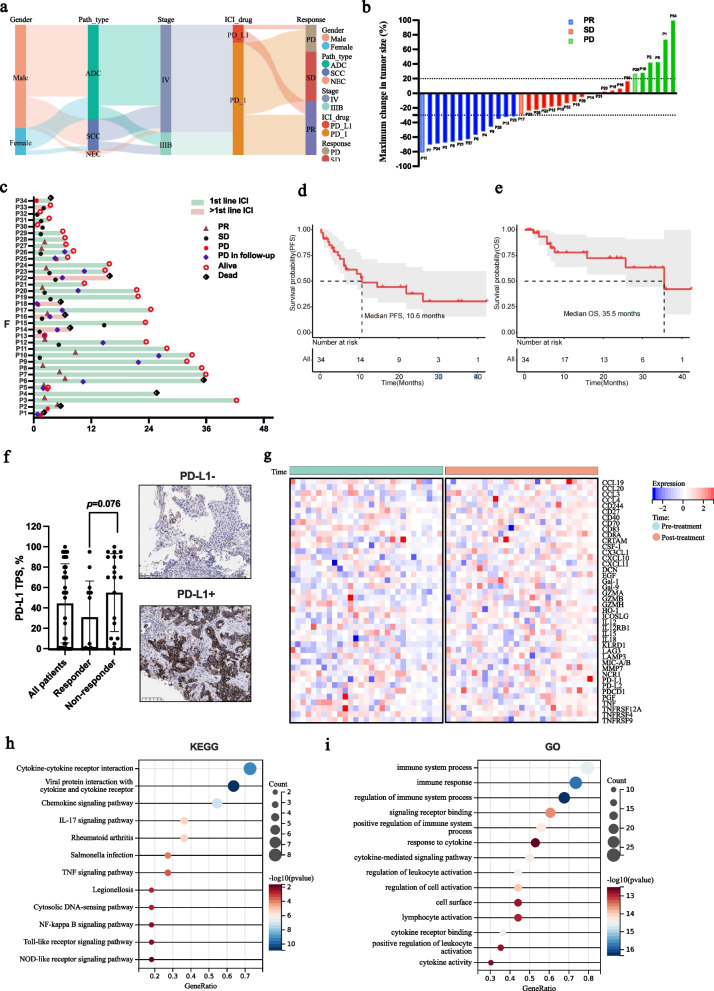
Patient feature, treatment response, survival, PD-L1 expression and plasma protein dynamics after ICI treatment in Cohort 1. **a** Patient feature in this study. **b** Waterfall chart of response to ICI therapy. PR, partial response; SD, stable disease; PD, progressive disease; **c**. Swimmer plot of interval of ICI treatment; **d**. Progression-free survival (PFS) curve; **e**. Overall survival (OS) curve of enrolled patients. **f** Histogram and IHC of tumor PD-L1 expression of enrolled patients; **g**. Heatmap of dynamic change of plasma protein after ICI therapy; **h**. KEGG analysis of upregulated proteins after ICI treatment; **i** GO analysis of regulated proteins after ICI treatment. ICI, immune checkpoint inhibitor

Various ICIs including pembrolizumab (*n* = 2), Camrelizuma (*n* = 11), Sintilimab (*n* = 12), Tislelizumab (*n* = 3), Atezolizumab (*n* = 1) and TQB2450 (an anti-PD-L1 antibody, *n* = 5) were utilized among patients in Cohort 1. After ICI treatment, 13 cases obtained partial response (PR) and the overall remission (OR) rate was 38.2% (Fig. 2a-b). Besides, 15 cases got stable disease (SD, 44.1%) and 6 cases had progression of disease (PD, 17.6%). Till the last visit in July 2024, the median follow-up was 15.4 (range, 0.82–42.3) months. A total of 19 cases underwent disease progression (54.2%) and 9 cases dead of lung cancer (25.7%, Fig. 2c). Median progression-free survival (PFS) and overall survival (OS) were 10.6 and 35.5 months, respectively (Fig. 2d-e). During treatment, 12 patients (PD-1 blockade, *n* = 10; PD-L1 blockade, *n* = 2) developed irAE involving respiratory (*n* = 7), hepatic (*n* = 1), cardiovascular (*n* = 2), renal (*n* = 1) systems and skin (*n* = 4; Table S2). ICI therapy was interrupted in 3 cases due to severe adverse reactions. Trational clinicopathological factors including PD-L1 presented limited values in predicting response to NSCLC immunotherapy in our study. No significant correlation was observed between treatment response and PD-L1 expression among patients in Cohort 1 (Fig. 2f). Univariate analysis revealed no significant clinicopathological factors related to PFS or OS neither (Table 2).

**Table 2:** Univariate cox-regression of survivals of NSCLC patients in Cohort 1

Characteristics	PFS		OS	
	**HR (95% CI)**	***p*-value**	**HR (95% CI)**	***p*-value**
**Gender, female *vs. male***	0.178 (0.023–1.347)	0.095	0.231 (0.030–1.785)	0.160
**Age, > 60* vs.* < 60 years**	2.484 (0.906–6.803)	0.077	0.836 (0.278–2.513)	0.750
**Smoking, yes* vs.* no**	0.981 (0.319–3.018)	0.973	1.447 (0.467–4.482)	0.522
**Pathology, ADC *vs.* others**	0.787 (0.317–1.955)	0.606	0.765 (0.265–2.214)	0.622
**Chemotherapy, yes *vs.* no**	0.695 (0.226–2.139)	0.526	0.704 (0.206–2.402)	0.575
**PD-L1, < 1% *vs.* ≥ 1%**	0.944 (0.514–1.736)	0.854	1.126 (0.445–2.846)	0.802
**Stage, IV* vs.* IIIb**	0.909 (0.845–1.073)	0.766	0.189 (0.010–3.743)	0.274
**ICI, anti PD-1 *vs.* anti PD-L1**	1.074 (0.511–2.257)	0.850	0.848 (0.443–1.623)	0.619

Abbreviations: *ADC* Adenocarcinoma, *WT* Wild type, *MT* Mutation, *R* Response, *NR* None response, *ICI* Immune checkpoint inhibitor.

In Cohort 2, 23 cases received anti-PD-L1 blockades and 7 cases had anti-PD-1 blockade therapy. The PR, SD and PD rates were 46.6% (14/30), 46.6% (14/30) and 6.6% (2/30), respectively. Median PFS and OS were 10.6 and 13.2 months, respectively (Fig. S1). During treatment, 5 patients developed irAE including immune-mediated pneumonitis (*n* = 3), dermatitis (*n* = 2), and hypocortisolism (*n* = 1).

### Dynamic changes of plasma proteins before and after ICI therapy for NSCLC

We investigated the plasma protein alternating regardless of response to ICI treatment based on the sequencing results of 58 paired plasma samples at baseline and after 2 cycles of ICI treatment from patients in Cohort 1. It was found that 42 out of 92 analyzed plasma proteins significantly upregulated after ICI treatment (*p* < 0.05; Fig. 2g). Function enrichment and signaling pathway analyses were performed based on the GO and KEGG databases to further explore the biological functions of the changing proteins. According to KEGG terms, up-regulated proteins were enriched in cytokine-cytokine receptor interaction pathway, viral protein interaction with cytokine and cytokine receptor, and chemokine signaling pathway (Fig. 2h). Several signaling pathways including immune system process, immune response, regulation of immune system process, signaling receptor binding and positive regulation of immune system process were enriched in GO set (Fig. 2i).

### Stratified analysis of plasma proteins based on patients’ response to ICI treatment

**Fig. 3:**
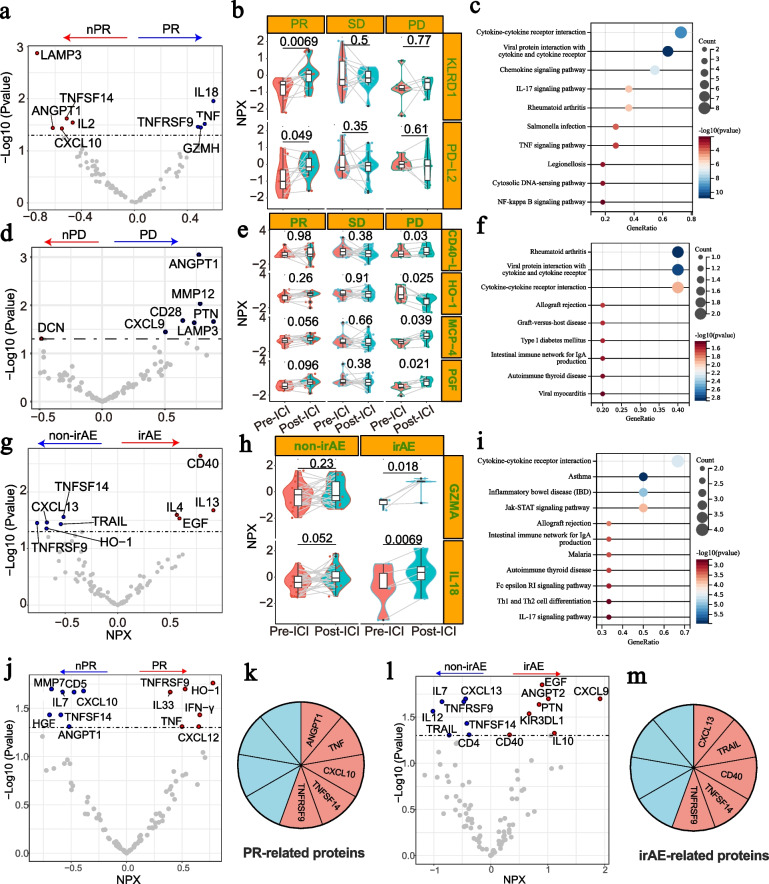
Plasma proteomics according to ICI therapy response and toxicity. **a**, **d**, and **g** The volcano plots represent the differentiated pre-treatment proteins between patients with PR and nPR (**a**), PD and nPD (**d**) and irAE and none irAE (**g**) in Cohort 1; **b, e **and** h**. The changed proteins after ICI treatment with predictive value to PR (**b**), PD (**e**) and irAE (**h**) in Cohort 1; **c, f **and** i**. KEGG analysis of differentiated proteins in patients with PR (**c**), PD (**e**), and irAE (**h**) from ICIs in Cohort 1; **j**. Validation of response-predictive proteins in Cohort 2; **k**. Consistence analysis of response-predictive proteins of the study and validation cohort; **l**. Validation of irAE-predictive proteins in Cohort 2; **m**. Consistence analysis of irAE-predictive proteins of the study and validation cohort. NPX, normalized protein expression; PR, partial response; nPR, none partial response; PD, progressive of disease; irAE, immune related adverse effect; ICI, immune checkpoint inhibitor

We further explored pre-treatment as well as changed proteins during treatment in patients with different responses to ICIs in Cohort 1 (Fig. 3). For patients who got PR, 4 pre-treatment plasma proteins (IL18, TNF, TNFRSF9 and GZMH) were significantly upregulated and 5 proteins were downregulated (LAMP3, IL2, ANGPT1, TNFSF14 and CXCL10; Fig. 3a) compared with those who got non-PR (nPR). Besides, the increased levels of KLRD1 and PD-L2 after treatment were associated with better ICI response (Fig. 3b). KEGG enrichment analysis revealed that PR-related proteins were involved in cytokine-cytokine receptor interaction pathway, viral protein interaction with cytokine and cytokine receptor, and chemokine signaling pathway (Fig. 3c). Besides, we investigated the proteins predictive of PD to ICI treatment. Results showed that the higher pre-treatment levels of CD28, LAMP3, CXCL9, ANGPT1, PTN and MMP12 were significantly associated with PD (Fig. 3d). Meanwhile, the elevation of CD40L, MCP4 and PGF, and the decrease of HO-1 in plasmas after ICIs indicated inferior therapeutic reactions (Fig. 3e). PD-related proteins were enriched in rheumatoid arthritis, viral protein interaction with cytokine and cytokine receptor, and cytokine and cytokine receptor pathways (Fig. 3f).

A total of 30 pre-treatment plasmas samples from patients in Cohort 2 were detected for validation of response-predictive proteins of ICI therapy (Fig. 3j-m). Results showed that 5 (ATGPT1, TNF, CXCL10, TNFSF14 and TNFRSF9) out of 9 response-predictive proteins found in Cohort 1 were also validated in Cohort 2 (Fig. 3j-k).

### Analysis of plasma proteins based on irAE development during ICI treatment

The utility of plasma proteins in irAE prediction was also explored in this study. In Cohort 1, those who developed irAE during ICI treatment were found to have higher pre-treatment levels of CD40, IL-4, IL-13 and EGF, but lower levels of CXCL13, TNFRSF9, TNFSF14, HO-1 and TRAIL in plasma (Fig. 3g). Meanwhile, IL-18 and GZMA elevations in plasma after ICI treatment were much more commonly documented in cases with irAE rather than those with none-irAE (Fig. 3h). Apart from cytokine-cytokine receptor interaction signaling pathway, JAK-STAT signaling pathway was also enriched based on irAE-related plasma proteins (Fig. 3i). Noteworthy, pre-treatment plasma CXCL13, CD40, TRAIL, TNFSF14 and TNFRSF9 were also validated as indicators of irAE for NSCLC patients in Cohort 2 (Fig. 3l-m).

Since patients harboring EGFR mutations have distinct microenvironment compared with driver gene negative patients, we compared the pre-treatment plasma proteins between them. No significant difference on plasma proteins between the EGFR-mutant and wild type patients was found (Fig. S2). However, this result should be interpreted cautiously since the number of driver gene positive patients was quite small in this study.

### Establish and evaluation of a plasma-based risk score for survival of ICI therapy

**Fig. 4:**
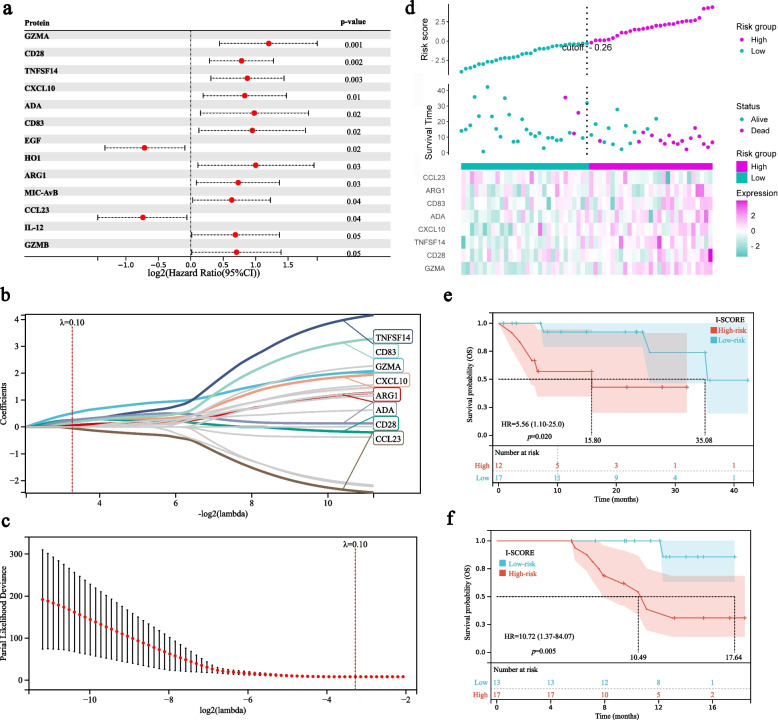
Cox-regression analysis and establish of a risk score model for overall survival for NSCLC patients receiving ICI therapy. **a** Forest plot of univariate survival analysis of pre-treatment plasma proteins. **b** LASSO coefficient profiles of candidate variables: **c**. Cross-validation curve for tuning parameter (λ) selection. **d** Establish of Immunosuppressive Signature of Combined Resistance Elements (I-SCORE) model based on eight prognostic proteins (CCL23, ARG1, CD83, ADA, CXCL10, TNFSF14, CD28 and GZMA) based on LASSO-COX regression. **e.** Stratification by I-SCORE of OS in the study cohort (Cohort 1); **f**. Stratification by I-SCORE of OS in the validation cohort (Cohort 2). I-SCORE, Immunosuppressive Signature of Combined Resistance Elements; ICI, immune checkpoint inhibitor

We further investigated the associations between plasma proteins and survival outcomes in NSCLC patients receiving ICI treatment. Through univariate Cox analysis, 9 proteins (GZMA, CD28, TNFSF14, CXCL10, ADA, CD83, HO-1, ARG1, and MIC-AvB) were identified to be associated with inferior OS, whereas 2 proteins (EGF and CCL23) were associated with superior OS (Fig. 4a). Subsequently, we enrolled the prognostic proteins with *p* < 0.10 in the LASSO-Cox regression analysis to avoid overfitting the model. Finally, a prognostic risk model was constructed by 8 plasma proteins by LASSO-Cox regression, including ARG1, CCL23, TNFSF14, CXCL10, CD28, TNFRSF9, GZMA, ADA and CD83 (Fig. 4b). These proteins were involved in T-cell excessive activation, exhaustion, and immunosuppression, the risk model was therefore named Immunosuppressive Signature of Combined Resistance Elements (I-SCORE). The optimal lambda was identified as 0.1 by the cross-validation analysis (Fig. 4c). According to I-SCORE model, patients were stratified into high-risk group and low-risk group with the cutoff value of -0.26 (Fig. 4d). High-risk group patients had significant inferior OS compared with those in the low-risk group in both the study cohort (5.8 *vs.* 35.2 months, *p* = 0.021; Fig. 4e) and the validation cohort (10.5 *vs.* 17.6 months, *p* = 0.005; Fig. 4f).

**Fig. 5:**
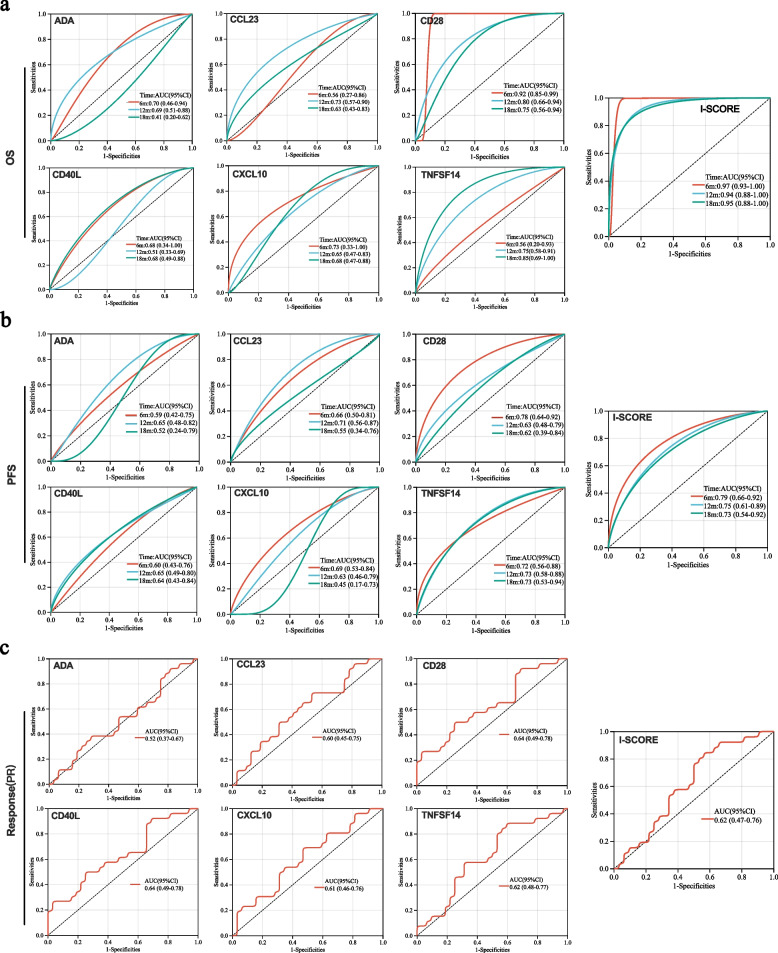
The predictive value of I-SCORE and single factors for OS, PFS and response in NSCLC. **a** Comparison of time-dependent ROC curves for OS using I-SCORE and single factor; **b**. Comparison of time-dependent ROC curves for PFS using I-SCORE and single factor; **c**. Comparison of ROC curves for response using I-SCORE and single factor. PFS, progression-free survival; OS, overall survival; I-SCORE, Immunosuppressive Signature of Combined Resistance Elements

We next performed time-dependent Receiver Operating Characteristic (ROC) curve analysis according to these separated predictive proteins and I-SCORE. It was found that single proteins could obtain a good predictive effect for OS with Area Under the ROC Curve (AUC) ranging from 0.56 to 0.92 for 6-month OS, from 0.51 to 0.80 for 12-month OS, and from 0.41 to 0.85 for 18-months OS, respectively. However, I-SCORE achieved significantly better predictions with 6, 12 and 18-month AUCs reaching 0.97 (0.93–1.00), 0.94 (0.93–1.00) and 0.95 (0.93–1.00), respectively (Fig. 5a). We also evaluated the prediction value of single proteins and I-SCORE for PFS. Results showed that I-SCORE model also presented satisfactory predictive effect for PFS with 6, 12, and 18-month AUCs of 0.79 (0.66–0.92), 0.75 (0.61–0.89) and 0.73 (0.54–0.92), respectively. Similarly, single factors presented limited values than I-SCORE model for PFS prediction (Fig. 5b).

Importantly, this risk model also had a modest predictive effect on therapeutic response of NSCLC patients. The AUC of the I-SCORE for predicting ICI response reached 0.62, which was similar to the predictive efficacy of single plasma proteins for treatment response (0.52–0.64; Fig. 5c).

### I-SCORE was associated with pro-inflammation and immunosuppression of NSCLC

**Fig. 6:**
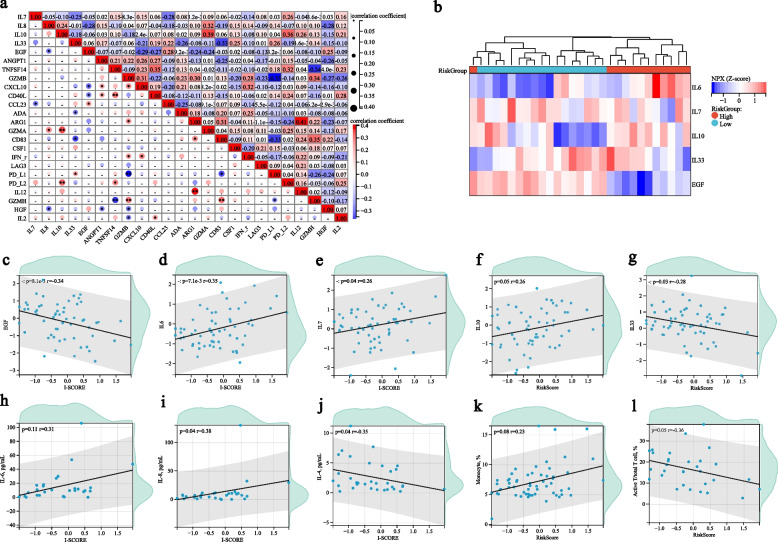
Correlation analysis of plasma proteins, I-SCORE, inflammatory and immunosuppressive factors. **a** Correlation matrix plot of certain plasma proteins in this study; **b**. Heatmap of inflammation and immunosuppression-related factors according to I-SCORE risk stratification; **c-g**. Correlation analysis of I-SCORE and inflammation and immunosuppression-related related factors based on Olink PEA; **h-j**. Correlation analysis of I-SCORE and inflammation related cytokines based on plasma ELISA; **k**. Positively linear correlation between I-SCORE and monocyte proportion; **l**. Negatively linear correlation between I-SCORE and active T cells in peripheral. I-SCORE, Immunosuppressive Signature of Combined Resistance Elements

We then investigated the association of plasma proteins and the I-SCORE. Apart from correlations within these predictive proteins, we also found correlations between the predictive factors and some interleukins (ILs), including IL-2, IL-7, IL-8, IL-10, IL-12 and IL-33 (Fig. 6a). Notably, patients in the high-risk group presented as a pro-inflammation and immunosuppressive feature with higher levels of pro-inflammatory cytokines IL-6, IL-7 and IL-10 and lower levels of immune stimulatory factors IL-33 and EGF (Fig. 6b). In addition, correlation analysis revealed that IL-6, IL-7, IL-10 were strong I-SCORE correlation proteins, whereas EGF and IL-33 were negative correlated factors (Fig. 6c-g).

To further validate this result, we detected the inflammation and immune-related cytokines in pre-treatment plasma using ELISA among enrolled patients. A moderate positive linear correlation between I-SCORE and plasma IL-8 levels (*r* = 0.38, *p* = 0.04; Fig. 6i), and a positive linear correlation was found between I-SCORE and plasma IL-4 levels (*r*=0.35, *p*=0.04; Fig. 6j). IL-6 also presented a positive linear correlation to I-SCORE, though the significance did not reach statistical difference (*r* = 0.31, *p* = 0.11; Fig. 6h). What’s more, high-risk patients were associated with higher proportions of monocyte (*r* = 0.23, *p* = 0.08; Fig. 6k) but decreased active T cell proportions in peripheral (*r* = 0.36, *p* = 0.05; Fig. 6l) compared to low-risk patients. These results suggested that the I-SCORE might indicate or reflect the hyperinflammation and immunosuppressive feature of patients with NSCLC. Targeting these proteins, or the hyperinflammation and immunosuppressive environment might be potential in facilitating ICI therapy efficacy and dismissing toxicity.

## Discussion

In this prospective exploratory study, we used the Olink Immuno-Oncology panel to explore potential plasma biomarkers for the prediction of ICI efficacy, toxicity, and survival outcomes in NSCLC. By analyzing 92 Immuno-Oncology related proteins, we identified 42 plasma proteins significantly elevating after ICI treatment which were enriched in cytokine-cytokine receptor interaction, chemokine signaling and immune response pathways. In addition, we identified several proteins with good predictive value in ICI response, including ATGPT1, TNF, CXCL10, TNFSF14 and TNFRSF9, as well as some irAE indicators (CXCL13, CD40, TRAIL, TNFSF14 and TNFRSF9). Furthermore, using LASSO-Cox regression model, we established an I-SCORE based on eight prognostic proteins in plasma. The I-SCORE stratified beneficial and non-beneficial patients from ICI treatment with satisfactory AUCs for OS, PFS and response. Besides, I-SCORE was correlated with excessive T cell activation, exhaustion, and the inflamed and immunosuppressive status which may lead to ICI therapy resistance. Targeting certain prognostic protein, or the hyperinflammation and immunosuppression might improve therapeutic effectiveness and long-term persistence of immunotherapy.

Current biomarkers for ICI therapy across multiple cancer types include PD-L1 expression, TMB, microsatellite instability (MSI) and tumor immune cell infiltration, all of which are related to the tumor and immune cells within the tumor microenvironment (TME).[22–27] However, most of these existing biomarkers have limited sensitivity or specificity and require biopsy samples. Due to the temporal and spatial heterogeneity of tumors and the dynamic nature of the TME, biopsies provide an incomplete representation of tumor and changes during treatment. To overcome these limitations, efforts are focused on the development of liquid biopsies for analyzing cell-free DNA (cfDNA), circulating tumor DNA (ctDNA), exosomes, microRNA (miRNA) and protein levels in peripheral blood as potential biomarkers.[28–30] High-throughput PEA from Olink, a methodology integrated the specificity of antibody-linked detection and the sensitivity of the polymerase chain reaction (PCR), enable the precise multiplex detection of low-abundance proteins using only microliter quantities of plasma.[31, 32] To the best of knowledge, this study was a prospective exploratory study composing a study cohort and a validation cohort using Olink PEA to better pinpoint the molecular change associated with ICI efficacy and toxicity from the peripheral perspective. Our study highlights the potential application of Olink plasma proteome as a liquid biopsy method for biomarker exploration in NSCLC patients receiving ICI therapy.

In a study of plasma proteome in esophageal cancer, 17 proteins including ADA, TNFSF14, ANGPT1, IL-7, IL-6, IL-8, EGF and CD40L were proved to be associated with unfavorable OS with ICI treatment (*p* < 0.05) [19]. Some of these results were consistent with parts of our present study in NSCLC, suggesting certain shared ICI resistance mechanisms between the two malignancies. It is noteworthy that some of the predictive or prognostic markers found in the present study have also been reported to harbor predictive value, mainly discovered by transcriptomic sequencing on tumor tissues. Of particular interest, some predictive proteins found in our study including CD28, TNFRSF9/41-BB, CXCL10 and GZMA were either co-stimulatory molecular for T cell activation, chemoattractant for T cell migration or component of T cell cytotoxicity in TME, whose expression are associated with improved T cell polyfunctionality, expansion, and differentiation [33–37]. Stimulation of these proteins improve the efficacy of CAR-T and ICI therapy in various cancers [33, 38–40]. However, the soluble forms of these proteins in plasma were proved as indicators of inferior efficacy in this study, suggesting the excessive activation and exhaustion of T cell function in these patients. In fact, only a few studies have examined the association between soluble immune checkpoint-related proteins and cancer outcomes. Soluble T cell regulatory proteins CD28, CD80, CTLA4, and HVEM were correlated with both biochemical recurrence and progression risks in prostate cancer [41]. Moreover, soluble CD28 and LAG3 were found negatively correlated with cytolytic activity of T cells in clear cell renal cell carcinoma (r = − 0.33 and − 0.32, respectively) [42]. Additionally, CD83 is an activation marker for antigen presenting cells, and soluble CD83 is reported elevated in the serum of patients with autoimmune disease and some hematological malignancies with an immune suppressive function [43]. Our study further confirmed these soluble molecules (CD28, CXCL10, TNFRSF9, TNFSF14, CD83, and HVEM) as predictive biomarkers in NSCLC, and might be potential targets to boost immunotherapy in patients.

ARG1 regulates the cancer cell immune escape through various manners in TME. Highly expressed ARG1 in cancer cells directly impairs T cells function by depleting L-arginine in TME [44]. Besides, secreted ARG1 from tumor-associated macrophages (TAMs) and myeloid-derived suppressor cells (MDSCs) depletes the L-arginine in TME [45]. ADA (adenosine deaminase) catalyze the transformation of adenosine into inosine. The ADA1 isoform, present extracellularly in plasma/serum, mediates T cell costimulation via binding to CD26 (its cognate receptor on T lymphocytes).[46, 47] CCL23, with both pro- and anti-cancer properties, is produced by eosinophils, monocytes and MDSCs activated by IL-1β [48]. In liver cancer, CCL23 recruited CD8 + T cell infiltration and enhancing the efficacly of immune response.[49] This study, for the first time, reported the positive predictive value of CCL23 in NSCLC, which might due to the attraction of resting T lymphocytes in TME.[48] However, further studies are warrant in order to fully explore its function in cancer immunity.

Taken together, we established the I-SCORE based on these eight prognostic proteins including ADA, ARG1, CD28, CD83, TNFSF14, CXCL10, GZMA and CCL23. The integrated risk model showed better predictive value than single factors for OS, PFS and response for NSCLC. Furthermore, our study reported the I-SCORE was associated with pro-inflammation and immunosuppression of NSCLC TME (positively linear with IL-6, IL-8 and monocytes, and negative linear with activated T cells). It is widely accepted that chronic inflammation critically contributes to cancer and persistent inflammation status mediates tumor-induced tolerance [50]. IL-6 is one common pro-inflammatory cytokine and proved as a predictive biomarker and desensitizer of immunotherapy responses in NSCLC patients [51, 52]. Elevated neutrophil–lymphocyte ratio (NLR) is another reflection of imbalanced inflammation and immunity status, which is also a negative prediction for survival outcome and irAE [53–56]. Therapeutic manipulation of chronic inflammation (such as anti-IL-6 blockade and neutralizing IL-8) is therefore likely to enhance immunotherapy efficacy [56–58]. These results suggest I-SCORE as a better reflection of comprehensive cancer-immune interaction, and a potentially reliable biomarker for ICI therapy. Besides, our study provided us a hint that suggested that switching the chronic pro-cancer inflammatory environments into an anti-cancer milieu might attenuate anticancer activity.

Our study has several limitations. Firstly, this study has a long time-span which covered the prevalence of COVID19, thus the patient sample size was not large and the treatment was not identical among enrolled patients. Besides, dynamics of plasma proteins were not validated yet. These findings were exploratory which need to be further confirmed in additional large cohort in the future. In conclusion, dynamic liquid biopsy using plasma proteomics suggested a potential noninvasive approach in predicting response and tolerance of PD-1/PD-L1 blockades in NSCLC patients.

## Material and method

### Patient eligibility

Consecutive patients with inoperable locally advanced, metastatic or recurrent NSCLC from August 2020 to June 2023 at Beijing Chest Hospital were initially enrolled. Inclusion criteria were: pathologically diagnosed NSCLC; stage IIIB-IV or recurrent disease; patients who intended to receive antibody against PD-1/PD-L1 with/without systemic chemotherapy; at least one measurable lesion; patients who were willing to provide peripheral blood samples before and during treatment. Patients who failed EGFR or ALK-TKIs and underwent subsequent ICIs ± chemotherapy were also included. Patients with an autoimmune disease, previous immune checkpoint blockade therapies, active or untreated central nervous system metastases, incomplete clinicopathologic or follow-up information, or those refused to provide peripheral blood were excluded. Written informed consent was provided by all the patients before enrollment.

### Study design and treatment

The study schema was shown in Fig. 1. This exploratory study consisted two cohorts: Cohort 1 was the primary prospective cohort and Cohort 2 was the retrospective cohort for validation. Enrolled patients received ICIs ± systemic chemotherapy according to investigator’s choice and patient’s willingness. ICIs were administrated including anti-PD-1 (pembrolizumab, Camrelizuma, Sintilimab and Tislelizumab) and anti-PD-L1 blockades (Atezolizumab and TQB2450) at a 21-day interval. For those who received chemotherapy, four or six 21-day cycles of induction treatment were administrated. Patients with adenocarcinoma received PC regimen: pemetrexed at 500 mg/m^2^ IV (day 1) and carboplatin at an area under the concentration time curve of 5 mg/mL/min IV (day 1). For patients with other subtypes, 4 or 6 cycles of induction NC regimen were administrated (nab-paclitaxel at 130 mg/m^2^ IV, days 1 and 8 and carboplatin at an area under the concentration time curve of 5 mg/mL/min IV, day 1) followed by corresponding ICI as the maintenance treatment. For those receiving no chemotherapy, ICI was given at a 21-day interval till disease progression or intolerance.

### Response and safety assessment

Tumor assessment was performed at baseline, and response assessment was performed every 6 weeks during the induction phase and every 9 weeks thereafter until disease progression according to RECIST version 1.1. Efficacy evaluation included complete response (CR), partial response (PR), stable disease (SD), and progressive disease (PD). Response was defined as CR and PR. Non-responders were defined as patients who got SD and PD upon ICI treatment. The safety and tolerability of ICIs were evaluated according to National Cancer Institute Common Terminology Criteria for AEs Version 4.0.

### Sample collection

Cohort 1 plasma samples were collected with the ongoing of this prospective study. Pre-treatment plasma from each patient were collected before the initiation of ICI treatment (within 7 days prior to ICI delivery) and post-treatment plasma samples were collected after two cycles of treatment (before the 3rd cycle of therapy) from patients in Cohort 1. For patients in Cohort 2, plasma samples were collected from before ICI treatment. All plasma samples were stored at -80℃ refrigerator.

### Proximity extension assay (PEA)

Plasma protein levels for 92 targets (Table S1) were quantified using the Olink® Immune Oncology Panel (Olink Proteomics AB, Uppsala, Sweden), following the manufacturer's protocol based on Proximity Extension Assay (PEA) technology. This method employs matched pairs of oligonucleotide-conjugated antibodies that bind target proteins. When antibodies are in proximity, their oligonucleotides hybridize, enabling proximity-dependent DNA polymerization catalyzed by DNA polymerase to generate a unique PCR amplicon. Amplification and quantification were performed using a microfluidic real-time PCR system (Biomark HD, Fluidigm).

Data underwent normalization to generate Normalized Protein Expression (NPX) values, incorporating internal and external controls for quality assurance. Internal controls, spiked into all samples, monitor specific PEA stages: two incubation/immunoassay controls, one extension control, and one detection control. External controls comprised a negative control for determining the limit of detection (LOD) and triplicate interpolate controls (IPCs) for data normalization.

Quality control was performed hierarchically: First, calculated standard deviation (SD) for detection and incubation/immunoassay controls. Runs required SD < 0.2 for acceptance. Then, evaluated detection and one incubation control against the run median. Samples exceeding ± 0.3 NPX from the median for both controls were excluded.

### Functional enrichment analyses

The differential plasma protein expressions before and after ICI treatment, of response and irAE were tested using the Wilcoxon rank-sum test. Proteins with a *p* < 0.05 were selected as subjects for enrichment analyses. The enrichment analysis was performed on the Kyoto Encyclopedia of Genes and Genomes (KEGG) as well as Gene Ontology Biological Process (GO) to categorize the biological functions of genes using the “cluster Profiler” package in R.

### Prognostic analysis and construction of I-SCORE

To further identify the prognostic plasma proteins, univariate Cox regression analysis was firstly applied to identify the prognostic variables for OS. Subsequently, variables with both log-rank and likelihood *p* < 0.10 on univariate analysis were enrolled in the LASSO-Cox regression analysis to avoid overfitting the model. The contraction penalty term (λ) was introduced into the model to optimize the regression model. Three-fold cross-validation was used to determine the optimal λ by choosing the logarithm of the minimum mean squared error of the lambda. LASSO-Cox-regularized regression analysis was performed using “glmnet” package in R. The formula of the risk score was as follows: RiskScore = 0.507722*GZMA + 0.258247*CD28 + 0.173803*TNFSF14 + 0.212909*CXCL10 + 0.248964*ADA + 0.157292*CD83 + 0.054064*ARG1-0.051968*CCL23. Patients were divided into high-risk and low-risk groups by the median risk score. Time-dependent ROC curves were plotted using the ‘survival ROC’ package to calculate AUC value at 6, 12, and 18 months of the risk score model.

### Flow cytometry and lymphocyte immunotyping

Peripheral blood monocyte cells (PBMCs) were isolated using density gradient centrifugation using 5 mL whole blood samples. Antigens targeted for immunophenotyping of PBMCs included HLA-DR, CD45, CD3, CD4, CD8, CD25, CD19, CD14, CD127, HLA-DR, CD16, and CD56. Samples were stained with fluorochrome-conjugated monoclonal antibodies, followed by incubation at room temperature for 20 min. Flow cytometric analysis was performed using a Beckman Coulter instrument (Brea, CA, USA) to quantify the following cell populations: B cells (CD3^−^CD19^+^), T cells (CD3^+^), T helper cells (Th, CD3^+^CD4^+^), T cytotoxic cells (Tc, CD3^+^CD8^+^), T regulatory cells (Treg, CD4^+^CD25^+^CD127^low^), NK cells (CD3^−^CD16^+^CD56^+^), and NKT cells (CD3^+^CD16^+^CD56^+^). Active T cells were defined as HLA-DR^+^CD3^+^ T cells. The gating strategy was resented in Fig. S3.

### Plasma cytokine detection assay

Plasma cytokines were detected using a validated ELISA-based ProcartaPlex (Immuno-Oncology Checkpoint 14-Plex Human ProcartaPlex™ Panel 1; Invitrogen™). Quantify concentrations from standard curves (pg/mL).

### Targeted sequencing

Tumor genome DNA was extracted from tumor tissue using the QIAamp DNA FFPE Tissue Kit (Qiagen, Valencia, CA). DNA quantification was performed using a Qubit 2.0 fluorimeter (Thermo Fisher Scientific). Nextseq500 (Illumina, San Diego, CA) with paired-end reads was used for targeted next-generation sequencing of a panel of 60 lung cancer-related genes. Loci with a depth of < 100 were filtered out. Variants with a frequency of > 0.1% were categorized as SNPs and excluded.

### PD-L1 expression

Fresh or formalin-fixed paraffin-embedded (FFPE) tumor samples were stained with PD-L1 antibody (clone 22C3) following the manufacturer’s standard protocol (Dako 22C3 PharmDx Assay; Agilent, Santa Clara, CA). PD-L1 expression was determined using the tumor proportion score (TPS). TPS of 0, 1–49% and ≥ 50% were defined as negative, intermediate and high PD-L1 expression, respectively.

### Statistical analysis

Data analysis was performed using R software (version 4.0.0), and flow diagrams were drawn by FigDraw. Mean ± standard deviation (*x̅* ± *sd)* was used for description of data that were normally distributed. Pearson’s chi-square test or linear-by-linear association was used for crossover analysis of clinicopathological features. Comparisons of the average values between different patient group were analyzed using student’s t test. All reported *p* values are two-sided, and *p* values less than 0.05 were considered statistically significant. PFS was defined as time interval from the date of treatment to the date of progression, relapse, last follow-up or death from any cause. OS was defined as the time interval from the date of treatment to the date of death from any cause, or last follow up.

## Supplementary Information

Supplementary Material 1.

## Data Availability

All data were included in this paper and deposited online (https://github.com/zhangfan69/TCR-data).
