# Supplementary material for: Liquid biopsy using plasma proteomics in predicting efficacy and tolerance of PD-1/PD-L1 blockades in NSCLC: a prospective exploratory study
Source: Mol Biomed. 2025 Jul 15;6:51. doi: 10.1186/s43556-025-00291-6 (PMC12260144; doi:10.1186/s43556-025-00291-6)

**Liquid biopsy using plasma proteomics in predicting efficacy and tolerance of PD-1/PD-L1 blockades in NSCLC: a prospective exploratory study**

Yuan Gao^1,2#^, Fei Qi^1,2#^, Wenhao Zhou^3#^, Peng Jiang^1^, Mingming Hu^1,2^, Ying Wang^1,2^, Congcong Song^3^, Yi Han^4^, Dongdong Li^5^, Na Qin^6^, Hongmei Zhang^1,2^, Haitao Luo^3^, Tongmei Zhang^1,2*^, Hongxia Li^1,2*^

^1^General Department, Beijing Chest Hospital, Capital Medical University; Beijing Tuberculosis and Thoracic Tumor Research Institute, Beijing, China;

^2^Laboratory for Clinical Medicine, Capital Medical University;

^3^Shenzhen Engineering Center for Translational Medicine of Precision Cancer Immunodiagnosis and Therapy, YuceBio Technology Co., Ltd, Shenzhen, China;

^4^Cardiothoracic Surgery Department, Affiliated Hospital of Shanxi University of Chinese Medicine, Shaanxi University of Chinese Medicine, Shanxi, China;

^5^School of Life Sciences, Anhui Medical University, Anhui, China;

^6^Outpatient Department, Beijing Chest Hospital, Capital Medical University; Beijing Tuberculosis and Thoracic Tumor Research Institute, Beijing, China.

^*^Corresponding:

**Hongxia Li**, General Department, Beijing Chest Hospital, Capital Medical University; Beijing Tuberculosis and Thoracic Tumor Research Institute; Laboratory for Clinical Medicine, Capital Medical University, Beijing, China, 101149. Address: No.9 Beiguan Street, Tongzhou District, 100149, Beijing, China. Email: Doctor.hong@163.com.

**Tongmei Zhang**, General Department, Beijing Chest Hospital, Capital Medical University; Beijing Tuberculosis and Thoracic Tumor Research Institute; Laboratory for Clinical Medicine, Capital Medical University, Beijing, China, 101149. Address: No.9 Beiguan Street, Tongzhou District, 100149, Beijing, China. Email: tongmeibj@163.com.

^#^These authors contributed equally to this work and listed as co-first authors.

**Table S1 The Olink Immuno-Oncology panel using in this study.**

| ADA | ADGRG1 | ANGPT1 | ANGPT2 | ARG1 | CAIX | CASP-8 | CCL17 | CCL19 | CCL20 |
| --- | --- | --- | --- | --- | --- | --- | --- | --- | --- |
| CCL4 | CD244 | CD27 | CD28 | CD4 | CD40 | CD40-L | CD5 | CD70 | CD83 |
| CSF-1 | CX3CL1 | CXCL1 | CXCL10 | CXCL11 | CXCL12 | CXCL13 | CXCL5 | CXCL9 | DCN |
| FGF2 | Gal-1 | Gal-9 | GZMA | GZMB | GZMH | HGF | HO-1 | ICOSLG | IFN-gamma |
| IL12 | IL12RB1 | IL13 | IL15 | IL18 | IL2 | IL33 | IL4 | IL5 | IL6 |
| KIR3DL1 | KLRD1 | LAG3 | LAMP3 | LAP TGF-bet1 | MCP-1 | MCP-2 | MCP-3 | MCP-4 | MIC-A/B |
| MUC-16 | NCR1 | NOS3 | PD-L1 | PD-L2 | PDCD1 | PDGF subunit B | PGF | PTN | TIE2 |

**Table S2 Immune-related adverse events among patients in Cohort 1.**

| **ID** | **Sex** | **Age** | **Pathology** | **Stage** | **PD-L1, TPS, %** | **IrAE** | **Time from ICIs, weeks** | **Treatment interruption** |
| --- | --- | --- | --- | --- | --- | --- | --- | --- |
| ID3 | Male | 73 | SCC | T2N0M1a | 55 | G1myositis, hepatitis and pneumonia | 9 | 0 |
| ID4 | Male | 74 | Aden | T4N2M1c | 0 | G1 myocarditis | 10 | 0 |
| ID8 | Female | 53 | Aden | T1N1M1c | 0 | G2 dermatitis | 7 | 0 |
| ID10 | Male | 69 | SCC | T2N3M1a | 0 | G2 pneumonia | 5 | 1 |
| ID11 | Female | 79 | Aden | T4N0M1a | 10 | G2 capillary hyperplasia | 9 | 0 |
| ID12 | Male | 74 | LCNEC | T4N0M1c | 80 | G1 nephritis，pneumonia | 6 | 0 |
| ID15 | Male | 76 | SCC | T1cN3M0 | 90 | G2 pneumonia | 6 | 1 |
| ID17 | Male | 64 | Aden | T4N3M0 | 0 | G1 capillary hyperplasia | 12 | 0 |
| ID13 | Male | 58 | Aden | T2N2M1b | 95 | G1 pneumonia | 7 | 0 |
| ID24 | Male | 73 | NSCLC | T4N2M1b | 20 | G1 pneumonia | 8 | 0 |
| ID26 | Male | 54 | Aden | T2N3M0 | 0 | G1 dermatitis | 6 | 0 |
| ID31 | Male | 65 | SCC | T2N1M1c | 100 | G2 pneumonia myocarditis | 10 | 1 |

Abbreviations: ICI=immune checkpoint inhibitor; PC=pemetrexed and carboplatin; NC=nab-paclitaxel and carboplatin; irAE=Immune-related adverse events; Aden=adenocarcinoma; SCC=squamous cell carcinoma.

**Fig. S1 Survival curves of patients in Cohort 2.**

**
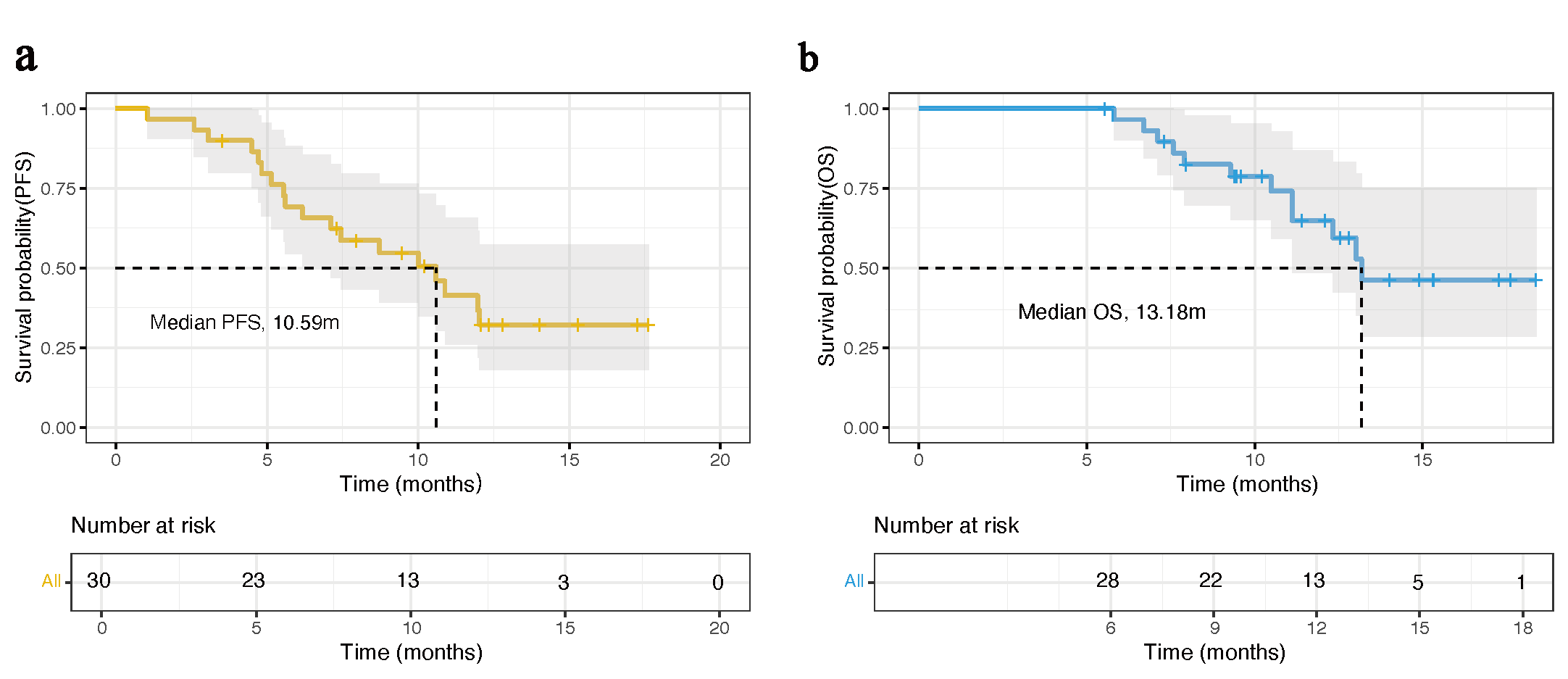
**

**Fig. S2** **Heatmap of DEPs classified by EGFR mutation status in NSCLC patients.**


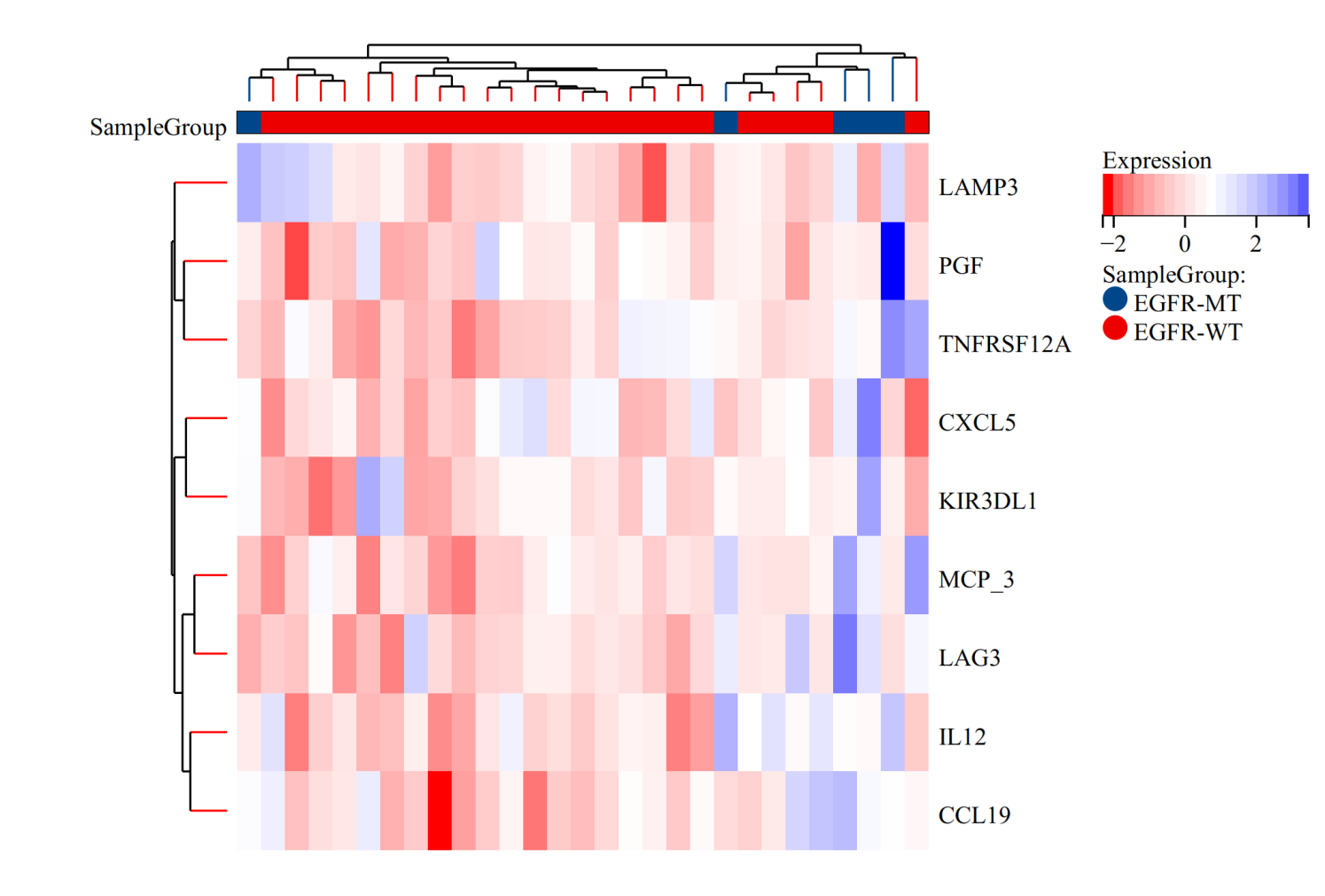


**Fig. S3 Gating strategy of immunophenotyping of peripheral cells.**


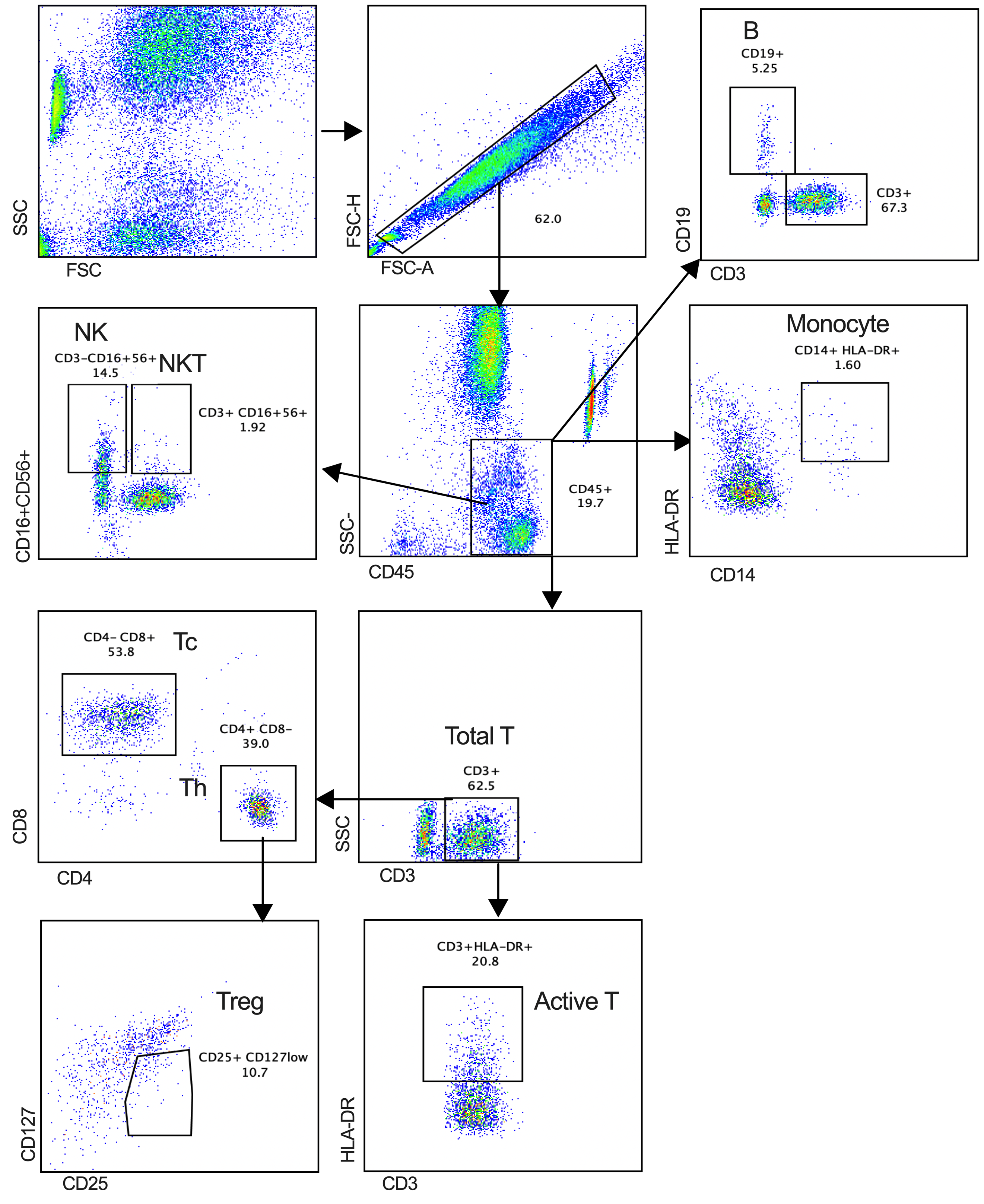

Supplement: Supplementary file 1 — Supplementary Material 1. [file 43556_2025_291_MOESM1_ESM.docx]
